# Impaired Verb-Related Morphosyntactic Production in Multiple Sclerosis: Evidence From Greek

**DOI:** 10.3389/fpsyg.2020.02051

**Published:** 2020-08-27

**Authors:** Valantis Fyndanis, Lambros Messinis, Grigorios Nasios, Efthimios Dardiotis, Maria Martzoukou, Maria Pitopoulou, Aikaterini Ntoskou, Sonia Malefaki

**Affiliations:** ^1^Center for Multilingualism in Society Across the Lifespan (MultiLing), University of Oslo, Oslo, Norway; ^2^Department of Rehabilitation Sciences, Cyprus University of Technology, Limassol, Cyprus; ^3^Neuropsychology Section, Departments of Psychiatry and Neurology, University Hospital of Patras and University of Patras Medical School, Patras, Greece; ^4^Department of Speech and Language Therapy, School of Health Sciences, University of Ioannina, Ioannina, Greece; ^5^Department of Neurology, University Hospital of Larisa, University of Thessaly, Larisa, Greece; ^6^Rehabilitation Unit for Patients with Spinal Cord Injury, “Demetrios and Vera Sfikas”, Department of Medicine, University of Patras, Patras, Greece; ^7^Department of Mechanical Engineering and Aeronautics, University of Patras, Patras, Greece

**Keywords:** relapsing-remitting multiple sclerosis, secondary progressive multiple sclerosis, morphosyntactic production, subject–verb agreement, time reference/tense, grammatical aspect, Greek

## Abstract

**Background:**

A recent systematic review found that language deficits are not very common in individuals with multiple sclerosis (MS). However, there are significant gaps in our knowledge about language abilities in MS. For instance, morphosyntactic production has not been explored adequately thus far. This study investigated verb-related morphosyntactic production in MS focusing on Greek, a morphologically rich language.

**Methods:**

A sentence completion task tapping into the production of subject–verb agreement, time reference/tense, and grammatical aspect was administered to 39 Greek-speaking individuals with MS [25 individuals with relapsing-remitting MS (RRMS group) and 14 individuals with secondary progressive MS (SPMS group)]. The task included only regular verbs. Generalized linear mixed-effects models were used to investigate the ability of individuals with MS to produce the above-mentioned morphosyntactic categories.

**Results:**

Overall, the RRMS and SPMS groups performed significantly worse than their matched control groups. Moreover, all four groups performed significantly worse on grammatical aspect than on subject–verb agreement and time reference. The difference between subject–verb agreement and time reference was not significant in any of the four groups. The overall performances of the RRMS and SPMS groups did not differ significantly.

**Conclusion:**

Individuals with MS are impaired in verb-related morphosyntactic production. Moreover, the pattern of performance of individuals with MS is identical to that exhibited by neurologically healthy individuals. Thus, the production performance of individuals with MS on verb inflection differs from that of healthy controls quantitatively but not qualitatively.

## Introduction

Multiple sclerosis (MS) is an autoimmune, inflammatory, and neurodegenerative disease of the central nervous system that predominantly affects sensorimotor and physical abilities. Cognitive deficits affecting working memory (WM), long-term memory, executive functioning, attention, processing speed, and visuospatial perception are also common in MS (e.g., Rao, 1995; Messinis et al., 2018; Ntoskou et al., 2018; Brochet and Ruet, 2019), with deficits in WM and processing speed being more frequent and severe in secondary progressive MS (SPMS) than in relapsing-remitting MS (RRMS) (Brochet and Ruet, 2019, and references therein). Underlying mechanisms of cognitive impairment in MS include tissue damage, atrophy especially of gray matter (and, more specifically, atrophy of brain loci such as the thalamus, hippocampus, and putamen), and altered connectivity and synaptopathy (Nasios et al., 2020). Regarding language abilities in MS, the evidence is still inconclusive. A recent systematic review (Renauld et al., 2016) showed that “language impairments are possible but not very common in MS patients” (p. 110). Nevertheless, El-Wahsh et al. (2020) found that 75% of a sample of persons living with MS self-reported a language impairment (with the most affected language domains being word retrieval and/or confrontational naming, expressive language, and receptive language in spoken discourse). Importantly, these persons experienced a reduced quality of life than did those without language impairment (El-Wahsh et al., 2020). Several studies reported evidence that word (verb and noun) finding problems are common in MS (e.g., Sepulcre et al., 2011; Kambanaros et al., 2017; Brandstadter et al., 2019). Brandstadter et al. (2019) found that word-finding difficulties attested in MS are linked to left parietal cortical thinning. It should be noted, however, that there are still gaps in our knowledge regarding both the neural substrate of language impairment in MS and the aspects of language affected by MS. Morphosyntactic production, for example, has not been explored adequately thus far. This study investigates verb-related morphosyntactic abilities in MS focusing on Greek, a highly inflected language. In particular, we focus on subject–verb agreement, time reference/tense, and grammatical aspect.

### Background Information on Subject–Verb Agreement, Time Reference/Tense, and Grammatical Aspect^1^

In many languages, including Greek, the person and the number of the grammatical subject of a sentence are morphologically marked on the verb (subject–verb agreement). For example, in the English sentence *This woman walks to work every day*, the verb’s inflectional morpheme –*s* expresses the fact that the grammatical subject of the sentence (*this woman*) is in the singular number and third person. The Greek verb morphologically encodes the combination of three persons (first, second, and third person) and two numbers (singular and plural number) in all tenses.

Morphosyntactic/morphosemantic categories such as tense and grammatical aspect are also instantiated in verb morphology. Tense is one of the linguistic means of referring to different time frames (e.g., past, present, and future). Tense, which is encoded in the verb, locates an event in time. The most common tenses are present, past, and future. In Greek (and in many other languages), the present tense usually locates an event as simultaneous with the speaking time, past tense locates it prior to the speaking time, and future tense locates it subsequently to the speaking time. Time reference, which is closely related to tense, is a semantic category. Time reference is the “semantic counterpart” of tense because, in many languages including Greek, time reference is made through tenses. It should be noted, however, that there is no one-to-one correspondence between tense and time reference, as different tenses may refer to the same time frame. For instance, despite their semantic differences, both simple past tense and present perfect refer to the past. Moreover, a given tense may refer to more than one time frame. For example, present tense in Italian (e.g., *mangio* “eat”) refers to the present (e.g., *Adesso mangio pasta* “Now I eat-PRESENT.1st.SG pasta”), but it may also be used to refer to the future in the presence of a time adverbial referring to the future (e.g., *Domani mangio pasta* “Tomorrow eat-PRESENT.1st.SG pasta”).

While tense and time reference refer to *when*, grammatical aspect refers to *how*. For example, although the sentences *Yesterday I was singing the song “New York, New York” when Mary gave me a ring* and *Yesterday I sang the song “New York, New York”* refer to the past, they reflect two different ways of viewing the singing event. In the former sentence, the speaker views the singing event as progressive; in the latter sentence, the speaker views the singing event as non-progressive. This is an *aspectual* difference. The most important aspectual distinction is that between *perfectivity* and *imperfectivity*. A speaker uses the perfective aspect when they view an event as a whole, without focusing on the various separate phases making up that event. On the other hand, a speaker chooses to use the imperfective aspect, when they focus on the internal structure of the event. Thus, the difference in the way the singing event is seen in the sentences *Yesterday I was singing the song “New York, New York” when Mary gave me a ring* and *Yesterday I sang the song “New York, New York”* reflects the aspectual distinction between *imperfectivity* and *perfectivity*, respectively. Note that the progressive aspect is subsumed into the imperfective aspect. As one can infer from the examples above, seeing an event as in progress or as completed often depends on the speaker’s point of view. For this reason, grammatical aspect is considered to be a subjective category. The opposite appears to be the case for tense and subject–verb agreement.

### Previous Studies on Agreement, Time Reference, and Aspect—Predictions for Greek Multiple Sclerosis

As shown in *Background Information on Subject–Verb Agreement, Time Reference/Tense, and Grammatical Aspect*, subject–verb agreement, time reference, and grammatical aspect have intrinsically different (linguistic) properties. These different properties are likely to make differential demands to the speaker’s processing system. Based on findings of studies on healthy aging and different pathologies, which will be presented later in this section, it appears that grammatical aspect, the most demanding category, taxes the speaker’s processing system, and individuals with less efficient processing systems fare worse on the production of aspect than on the production of subject–verb agreement, which seems to be an undemanding morphosyntactic relation. This pattern seems to result from the combination of the differential demands that different verb-related morphosyntactic categories pose on the speaker’s processing system and a sufficiently large variability in processing efficiency across healthy and neurologically affected participants. Variability in processing efficiency is presumably reflected in variability in WM capacity or closely related constructs such as processing speed (e.g., Salthouse, 1992; Fry and Hale, 1996, 2000).

This statement gains empirical support from Greek Alzheimer’s disease (AD), stroke-induced aphasia, and healthy aging. Fyndanis et al. (2013) found that, in morphosyntactic production in Greek AD, grammatical aspect is more impaired than time reference/tense and subject–verb agreement, and time reference is more impaired than subject–verb agreement.^3^ Interestingly, Fyndanis et al. (2012, 2018b) reported the same pattern for Greek stroke-induced aphasia and healthy aging, while Nanousi et al.’s (2006) and Varlokosta et al.’s (2006) groups of Greek-speaking individuals with aphasia also fared significantly worse on the production of grammatical aspect than on the production of subject–verb agreement. That the same pattern emerged not only in AD and aphasia but also in healthy speakers (e.g., Fyndanis et al., 2018b) is consistent with the idea that pathology only exacerbates patterns or trends observed in healthy speakers (e.g., Miyake et al., 1994; Dick et al., 2001). If our participants with MS reveal a pattern of performance similar to that reported for Greek mild AD, aphasia and healthy aging, this finding would lend further empirical support to the idea that, at least in verb-related morphosyntactic production, pathology exacerbates trends or patterns exhibited by healthy speakers.

Fyndanis et al. (2012, 2013) accounted for the patterns of morphosyntactic performance that emerged in Greek aphasia and AD by employing the Interpretable Features’ Impairment Hypothesis (IFIH) (see also Varlokosta et al., 2006, and Nanousi et al., 2006). Framed within the minimalist program (e.g., Chomsky, 1995, 2000, 2001), the IFIH states that functional/morphosyntactic categories bearing interpretable features, such as aspect and tense/time reference, are more prone to impairment than categories bearing uninterpretable features (e.g., subject–verb agreement), because the former involve processing and integration of information from two distinct levels of representation (grammatical and extralinguistic/conceptual), whereas the latter require implementation of grammatical knowledge only (Fyndanis et al., 2012, 2013). In other words, as per IFIH, it is the involvement of integration processes that renders morphosyntactic categories bearing interpretable features more demanding in terms of processing resources than categories bearing uninterpretable features. The idea that integration processes tax speakers’ processing system is shared—-explicitly or implicitly—-by many scholars (e.g., Hartsuiker et al., 1999; Avrutin, 2000; Kok et al., 2007; Yarbay Duman and Bastiaanse, 2009; Bastiaanse et al., 2011). It should be noted that, in light of new data from Greek AD, Fyndanis et al. (2018c) revised the IFIH suggesting that only morphosyntactic categories involving both integration processes and inflectional alternations (e.g., *walk*–*walked*) are prone to impairment in individuals with processing limitations.

Although the construct of processing resources has been linked to cognitive constructs such as WM and processing speed, it is still unknown what is the exact nature of processing resources involved in verb-related morphosyntactic production. In other words, there is still uncertainty on the exact cognitive mechanism that is critically involved in the production of demanding morphosyntactic categories such as grammatical aspect. Fyndanis et al. (2018b) reported data from 103 Greek-speaking healthy individuals and eight persons with agrammatic aphasia (PWAs), which showed that, in both groups, verbal WM capacity affected the production of grammatical aspect more than that of time reference and did not affect the production of subject–verb agreement at all. Importantly, it was verbal WM capacity that shaped the pattern of verb-related morphosyntactic production (grammatical aspect < time reference < subject–verb agreement) exhibited by the groups of aphasic participants and healthy individuals in Fyndanis et al. (2018b). This finding was taken as further empirical evidence for IFIH (Nanousi et al., 2006; Varlokosta et al., 2006; Fyndanis et al., 2012).^4^ However, Fyndanis et al.’s (2018b) participants were only tested with a sentence completion task tapping into verb-related morphosyntactic production and with complex span tasks measuring verbal WM capacity. Cognitive capacities closely related to WM such as processing speed (e.g., Salthouse, 1992; Fry and Hale, 1996, 2000) were not assessed. In a study on the production of subject–verb agreement and tense in Dutch agrammatic aphasia, Kok et al. (2007) accounted for their agrammatic patients’ pattern of performance by assuming WM limitations on the basis of indirect evidence only, that is, without having measured their participants’ WM capacity or any other cognitive capacity. The authors manipulated computation load by using a simple task that only required computation and production of verb inflection and a complex task that required both computation/production of verb inflection and constituent/word ordering. More agreement and tense errors (inflection errors) occurred in the complex task. The authors explained the effects of processing load “by assuming that less working memory capacity is available for the computation of verb inflection in the Order and Inflection Test than in the Inflection Test, due to the necessity of computing the correct word order in the first, but not in the second case” (Kok et al., 2007, p. 281). However, one could also explain the effects of processing load in terms of processing speed, as processing speed is closely related to WM (Fry and Hale, 1996, 2000), and individual differences in WM capacity largely reflect individual differences in processing speed (e.g., Salthouse, 1992). In fact, Leonard (1998) suggested that the morphosyntactic impairment found in children with specific language impairment is related to their processing speed. Moreover, Chatziadamou (2018) tested 80 middle-aged and older healthy Greek-speaking individuals with the same sentence completion task used by Fyndanis et al. (2018b) and found that processing speed significantly affected the production of all three morphosyntactic categories (grammatical aspect, time reference, and subject–verb agreement).

Regardless of the exact contribution of WM capacity and processing speed to processing resources involved in verb-related morphosyntactic production, we assume that both cognitive capacities are critically involved in the production of demanding morphosyntactic features/categories. Given that MS is characterized by WM and processing speed limitations (Brochet and Ruet, 2019) —-among other deficits—- we expect individuals with MS to perform worse than healthy controls on tasks tapping into verb-related morphosyntactic production. We also expect them to exhibit a pattern of performance identical or similar to that emerged in Greek AD (Fyndanis et al., 2013), aphasia (Fyndanis et al., 2012, 2018b), and healthy aging (Fyndanis et al., 2018b) (i.e., grammatical aspect < time reference < subject–verb agreement). Moreover, since individuals with SPMS have greater cognitive limitations than individuals with RRMS (Brochet and Ruet, 2019), we expect the former to be more impaired than the latter in morphosyntactic production.

## Materials and Methods

### Participants

Thirty-nine Greek-speaking individuals with MS took part in the study. There were 25 participants with RRMS and 14 participants with SPMS. Moreover, there were two groups of Greek-speaking neurologically healthy controls. The RRMS and SPMS groups were matched with control group 1 (*N* = 28) and control group 2 (*N* = 15), respectively, on age, education, and sex (see Table 1). The healthy individuals who made up the two control groups were selected from a large cohort of healthy individuals (*N* = 103) who had taken part in Fyndanis et al.’s (2018b) study. All these healthy individuals were administered the same linguistic task that was employed in the current study (see section “Experiment”).

**TABLE 1:** Participants’ demographic and cognitive profile.

	**RRMS**	**SPMS**	**Control Group 1 (RRMS)**	**Control Group 2 (SPMS)**	**RRMS vs. Control Group 1**	**SPMS vs. Control Group 2**	**RRMS vs. SPMS**
Age	43.0 (±8.4)	49.8 (±10.0)	43.0 (±5.2)	50.9 (±6.8)	Mann–Whitney *U* test, *U* = 347.5, *p* = 0.972	Two-sample *T*-test, *t*(27) = 0.364, *p* = 0.719	Two-sample *T*-test, *t*(37) = -2.269, *p* = 0.029^∗^
Education	13.8 (±3.9)	12.3 (±3.7)	14.6 (±2.8)	13.7 (±4.0)	Mann–Whitney *U* test, *U* = 388.500, *p* = 0.478	Two-sample *T*-test, *t*(27) = 0.986, *p* = 0.333	Wilcoxon rank-sum Test, *T* = 218, *p* = 0.204
Sex	13 female, 12 male	10 female, 4 male	19 female, 9 male	10 female, 5 male	Chi-square test, *χ*^2^ (1, *N* = 53) = 0.80, *p* = 0.370	Chi-square test, *χ*^2^ (1, *N* = 29) = 0.00, *p* > 0.995	Chi-square test, *χ*^2^ (1, *N* = 39) = 1.40, *p* = 0.317
Processing speed (SDMT)	35.2 (±12.6)	27.6 (±8.9)	N.A.	N.A.	N/A	N/A	Two-sample *T*-test, *t*(37) = 1.965, *p* = 0.057
Visuospatial STM (BVMT-R)	11.5 (±8.7)	13.6 (±6.9)	N.A.	N.A.	N/A	N/A	Two-sample *T*-test, *t*(37) = 0.384, *p* = 0.703
Verbal learning (GVLT)	51.0 (±14.2)	50.9 (±13.9)	N.A.	N.A.	N/A	N/A	Two-sample *T*-test, *t*(37) = 0.024, *p* = 0.981

*According to the normative data (stratified by age and educational level) reported in Messinis et al. (2020), Greek-speaking healthy individuals matched with the RRMS group on age and level of education scored 48.8 (±7.9) on SDMT; and Greek-speaking healthy individuals matched with the SPMS group on age and level of education scored 47.8 (±9.1) on SDMT. Furthermore, 79 Greek-speaking healthy individuals [48 females and 31 males; mean age = 37.2 (±10.6); mean education = 15.6 (±5.5)] reported in Polychroniadou et al. (2016) yielded the following scores on the three tests making up BICAMS: SDMT = 61.4 (±13.1); BVMT-R = 22.1 (±6.5); GVLT = 60.5 (±10.7). N.A., not available; N/A, not applicable; SDMT, Symbol Digit Modalities Test; STM, short-term memory; BVMT-R, Brief Visuospatial Memory Test-Revised; GVLT, Greek Verbal Learning Test; RRMS, relapsing-remitting multiple sclerosis; SPMS, secondary progressive multiple sclerosis.*

The diagnosis of MS was based on the McDonald revised criteria (for a detailed description of these criteria, see Polman et al., 2011). Participants’ demographic and clinical characteristics (age, years of formal education, sex, medication, duration and severity of the disease, depression levels, and fatigue) were recorded and evaluated. Patients’ inclusion criteria included (1) MS diagnosis by an experienced neurologist; (2) clinical evaluation based on the Expanded Disability Status Scale (EDSS) and disability level ranging from 0 to 5; (3) no history of other neurological disorders; (4) score on the Mini Mental State Examination (MMSE) (Folstein et al., 1975; Greek version of MMSE: Fountoulakis et al., 2000) greater than or equal to 24; (5) no history of major psychiatric disorders or psychotic symptoms (delusions, hallucinations); (6) being native speakers of Greek; (7) no presence of relapses or any change in EDSS score over the last 6 months before participation in the study; (8) normal or corrected vision and hearing; and (9) no alcohol abuse or abuse of illegal drugs or steroids. SPMS patients did not experience any relapses or MRI activity for at least 12 months prior to inclusion in the study.

To obtain more information on the cognitive profile of the MS participants, we also administered the Greek version of the Brief International Cognitive Assessment for MS (BICAMS) (Langdon et al., 2012; Polychroniadou et al., 2016). The BICAMS is a tool that has been validated and employed in many countries. The Greek version of BICAMS consists of three tests: the Symbol Digit Modalities Test (SDMT), which primarily taps into information processing speed; the Brief Visuospatial Memory Test-Revised (BVMT-R), which taps into visuospatial short-term memory (STM) and learning; and the Greek Verbal Learning Test (GVLT), which taps into verbal/word learning. The descriptions of SDMT, BVMT-R, and GVLT are provided in Supplementary Material S1.

The work described here has been carried out in accordance with the Code of Ethics of the World Medical Association (Declaration of Helsinki) for experiments involving humans. The study was approved by the Ethics Committee of the University Hospital of Ioannina, Greece. All participants gave written informed consent.

### Experiment

Participants were administered a sentence completion task [developed by Fyndanis et al. (2018b)] tapping into verb-related morphosyntactic production. This task consisted of 192 experimental items: 64 items tapped into subject–verb agreement, 64 into time reference, and 64 into grammatical aspect. In the agreement condition, person and number agreement were tested. None of the grammatical subjects included a conflict between grammatical and notional number (Humphreys and Bock, 2005); that is, words like *audience* (grammatically singular but conceptually plural) and *scissors* (grammatically plural but conceptually singular) were excluded. In the time reference condition, verbs referring to the past and future were elicited. The grammatical aspect condition targeted verbs encoding perfective and imperfective aspect. All three conditions were matched on sentence length.

Participants heard a source sentence (SS) and the beginning of the target sentence (TS). They were required to orally complete the TS by providing the missing verb phrase. Participants always had to produce a different form of the verb than that appeared in the SS. Examples of the subject–verb agreement, time reference, and grammatical aspect conditions are given in Table 2.

**TABLE 2:** Examples of source sentence-target sentence pairs testing subject–verb agreement, time reference, and grammatical aspect.

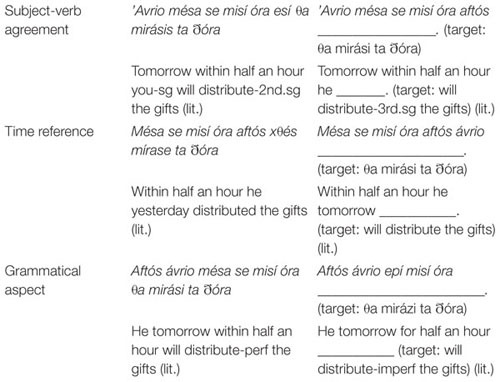

As can be seen in Table 2, the SSs differed from the TSs only in one feature value (person, number, time reference/tense, or grammatical aspect), conveyed by the subject or an adverbial, which was sufficient to elicit the target verb form associated with the morphosyntactic category tested by each item.

Sixteen bisyllabic regular transitive verbs (taking one grammatical object only) were used. All verbs were stressed on the penultimate syllable. Each of these verbs appeared six times in total, twice in each morphosyntactic condition (subject–verb agreement, time reference/tense, and grammatical aspect).

There were two lists. Each list consisted of 96 items: 32 items tested subject–verb agreement, 32 items tested time reference, and 32 items tested grammatical aspect. The same verbs appeared in both lists. Moreover, in each list, each verb appeared three times; it appeared once in each morphosyntactic condition. In each list, the three conditions were pseudorandomized such that there were never more than three consecutive items of the same condition. The item order (or presentation order) was kept constant for all participants. Each participant with MS was administered one list only, and the two lists were evenly distributed within the RRMS and SPMS groups. Healthy participants were administered both lists.

The dependent variable was *accuracy*. Scoring was based on the verb form produced by the participants. The grammatical object was not considered. Irrelevant morphosyntactic errors were ignored. For example, in the subject–verb agreement condition, time reference errors, or aspect errors were not considered.

### Data Analysis

We employed the lme4 package in R (Bates et al., 2014) and fitted generalized linear mixed-effects models (Pinheiro and Bates, 2000) to three datasets: (1) dataset of RRMS group and its matched control group; (2) dataset of SPMS group and its matched control group; and (3) unified dataset of RRMS and SPMS groups. Since accuracy was a dichotomous variable (1 = correct answer, 0 = wrong answer), the logistic model was used to model the probability of the correct answer (Jaeger, 2008). We fitted two models to each dataset. Model 1 included group and morphosyntactic condition as fixed effects; the interaction between group and morphosyntactic condition, subjects, and items as random effects; and morphosyntactic condition as by-subject random slope. Model 2 was identical to Model 1 except that it did not include the random slope. Model selection was based on the Akaike information criterion (see Burnham and Anderson, 2004). The best-fitting models (for the datasets above) are presented in Tables 3–5. Results are reported with two reference levels: subject–verb agreement and grammatical aspect. This enables us to compare all three levels of morphosyntactic condition (i.e., agreement, time reference, and aspect) to each other, as well as to check whether there were interactions between these three morphosyntactic levels and the two groups included in each dataset. We also checked whether there was an effect of list by fitting two generalized linear mixed-effects models to the unified MS dataset. Model 1 included list (two levels: List 1, List 2) and morphosyntactic condition as fixed effects; the interaction between list and morphosyntactic condition, subjects, and items as random effects; and morphosyntactic condition as by-subject random slope. Model 2 was identical to Model 1 except that it did not include the random slope. Again, model selection was based on the Akaike information criterion; the best-fitting model is presented in Supplementary Table S1.

**TABLE 3:** Generalized linear mixed-effects model on accuracy fitted to the dataset of the relapsing-remitting multiple sclerosis (RRMS) group and its matched control group.

**Term**	**Estimate**	**Std. error**	***z* Value**	**Pr(>| *z*|)**
Intercept (Group = Control Group; Morphosyntactic Cond. = Agreement)	7.430	0.802	9.263	< 0.001*
Morphosyntactic Cond. = Aspect	–2.992	0.786	–3.805	< 0.001*
Morphosyntactic Cond. = Time Ref.	3.143	1.815	1.732	0.083
Group = RRMS	–2.587	0.775	–3.337	< 0.001*
Morphosyntactic Cond. = Aspect: Group = RRMS	0.250	0.713	0.350	0.726
Morphosyntactic Cond. = Time Ref.: Group = RRMS	–1.930	1.517	–1.272	0.203
Intercept (Group = Control Group; Morphosyntactic Cond. = Aspect)	4.437	0.417	10.638	< 0.001*
Morphosyntactic Cond. = Agreement	2.992	0.794	3.769	< 0.001*
Morphosyntactic Cond. = Time Ref.	6.135	1.753	3.501	< 0.001*
Group = RRMS	–2.337	0.519	–4.505	< 0.001*
Morphosyntactic Cond. = Agreement: Group = RRMS	–0.250	0.717	–0.348	0.728
Morphosyntactic Cond. = Time Ref.: Group = RRMS	–2.179	1.451	–1.502	0.133

The model included the additive effect of morphosyntactic condition and group, the interaction between the two, subjects and items as random effects, and morphosyntactic condition as by-subject random slope. The symbol * indicates significant effects.

**TABLE 4:** Generalized linear mixed-effects model on accuracy fitted to the dataset of the secondary progressive multiple sclerosis (SPMS) group and its matched control group.

**Term**	**Estimate**	**Std. error**	***z* Value**	**Pr(>z|)**
Intercept (Group = Control Group; Morphosyntactic Cond. = Agreement)	7.535	0.862	8.741	< 0.001*
Morphosyntactic Cond. = Aspect	–3.522	0.745	–4.727	< 0.001*
Morphosyntactic Cond. = Time Ref.	0.699	1.232	0.567	0.570
Group = SPMS	–2.149	1.025	–2.097	0.036*
Morphosyntactic Cond. = Aspect: Group = SPMS	–0.601	0.852	–0.706	0.480
Morphosyntactic Cond. = Time Ref.: Group = SPMS	–1.432	1.326	–1.080	0.280
Intercept (Group = Control Group; Morphosyntactic Cond. = Aspect)	4.013	0.487	8.242	< 0.001*
Morphosyntactic Cond. = Agreement	3.522	0.748	4.711	< 0.001*
Morphosyntactic Cond. = Time Ref.	4.221	1.028	4.105	< 0.001*
Group = SPMS	–2.750	0.631	–4.361	< 0.001*
Morphosyntactic Cond. = Agreement: Group = SPMS	0.601	0.855	0.703	0.482
Morphosyntactic Cond. = Time Ref.: Group = SPMS	–0.831	1.071	–0.776	0.438

The model included the additive effect of morphosyntactic condition and group, the interaction between the two, and subjects and items as random effects. The symbol * indicates significant effects.

**TABLE 5:** Generalized linear mixed-effects model on accuracy fitted to the dataset of the relapsing-remitting multiple sclerosis (RRMS) and secondary progressive multiple sclerosis (SPMS) groups.

**Term**	**Estimate**	**Std. error**	***z* Value**	**Pr(>| *z*|)**
Intercept (Group = RRMS; Morphosyntactic Cond. = Agreement)	5.150	0.560	9.190	< 0.001*
Morphosyntactic Cond. = Aspect	–3.012	0.516	–5.843	< 0.001*
Morphosyntactic Cond. = Time Ref.	0.479	0.891	0.538	0.591
Group = SPMS	0.090	0.711	0.126	0.900
Morphosyntactic Cond. = Aspect: Group = SPMS	–0.954	0.573	–1.663	0.096
Morphosyntactic Cond. = Time Ref.: Group = SPMS	0.221	0.960	0.230	0.818
Intercept (Group = RRMS; Morphosyntactic Cond. = Aspect)	2.137	0.362	5.907	< 0.001*
Morphosyntactic Cond. = Agreement	3.012	0.516	5.843	< 0.001*
Morphosyntactic Cond. = Time Ref.	3.492	0.811	4.305	< 0.001*
Group = SPMS	–0.864	0.558	–1.549	0.121
Morphosyntactic Cond. = Agreement: Group = SPMS	0.954	0.574	1.662	0.096
Morphosyntactic Cond. = Time Ref.: Group = SPMS	1.174	0.916	1.282	0.200

The model included the additive effect of morphosyntactic condition and group, the interaction between the two, subjects and items as random effects, and morphosyntactic condition as by-subject random slope. The symbol * indicates significant effects.

## Results

**FIGURE 1:**
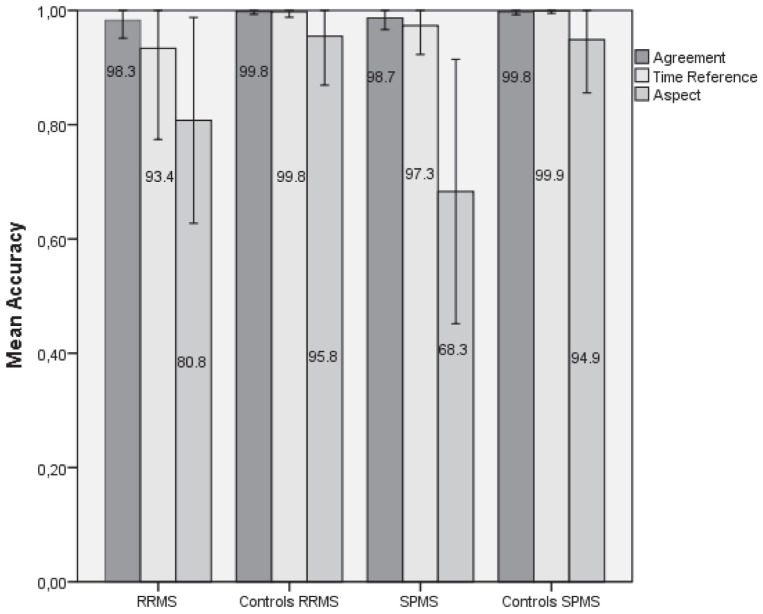
Percent performance and standard deviation of the four groups on the production of subject–verb agreement, time reference, and grammatical aspect.

As shown in Figure 1 and Tables 3, 4, the two control groups outperformed the corresponding MS groups on the sentence completion task, and all four groups fared worse on grammatical aspect than on subject–verb agreement and time reference/tense. The difference between agreement and time reference was not significant in any of the four groups. Moreover, as shown in Table 5, the RRMS group did not differ significantly from the SPMS group in overall performance. The overall performance of the unified MS group (i.e., group of all individuals with MS) on grammatical aspect, time reference and subject–verb agreement was 76.3, 94.8, and 98.4% correct, respectively, whereas the unified control group performed 94.7, 99.8, and 99.8% correct on grammatical aspect, time reference, and subject–verb agreement, respectively. List 1 and List 2 elicited similar performances (see Supplementary Table S1). As shown in Figure 1, there was a lot of variability in the aspect and time reference conditions within the RRMS group, and in the aspect condition within the SPMS group.

## Discussion

The overall performance of the RRMS and SPMS groups was significantly worse than that of the control groups, showing that individuals with MS can be impaired in verb-related morphosyntactic production. All four groups performed worse on grammatical aspect than on subject–verb agreement and time reference. The two MS groups, thus, differed from their control groups in morphosyntactic production quantitatively but not qualitatively. Contrary to our prediction, the RRMS group did not differ significantly from the SPMS group in any of the three morphosyntactic categories. This result might reflect the fact that the two MS groups did not differ significantly in cognitive abilities, as measured by BICAMS (Langdon et al., 2012; Polychroniadou et al., 2016) (see Table 1).

Results are consistent with the idea that pathology exacerbates patterns or trends observed in healthy speakers (e.g., Miyake et al., 1994; Dick et al., 2001; Fyndanis et al., 2018b). What follows from this idea is that patterns of morphosyntactic production are not pathology specific, meaning that the same or similar patterns should emerge in different neurological conditions. This prediction is largely borne out by the fact that the pattern reported here for MS is similar to the patterns reported for Greek aphasia (Fyndanis et al., 2012, 2018b) and AD (Fyndanis et al., 2013) in that, in all three neurological conditions, grammatical aspect was found to be more impaired than subject–verb agreement and time reference. The present results, however, differ from Fyndanis et al’s. (2012, 2013, 2018b) results from Greek-speaking aphasic and AD individuals in that, while these individuals performed significantly worse on tense/time reference than on subject–verb agreement, our MS participants fared comparably well on these two morphosyntactic categories. *Prima facie* this result is not consistent with IFIH (Nanousi et al., 2006; Varlokosta et al., 2006; Fyndanis et al., 2012), as this hypothesis states that all morphosyntactic categories bearing interpretable features (and thus involving integration processes) are more prone to impairment than categories bearing uninterpretable features (and thus not requiring integration processes). Unlike subject–verb agreement, both grammatical aspect and time reference/tense bear interpretable features and involve integration processes. As implicitly acknowledged by Fyndanis et al. (2012, 2013, 2018b), however, it appears that involvement of integration processes is not the only factor that taxes the speaker’s processing system. This is reflected in the patterns of performance that they reported: Greek-speaking PWAs and individuals with mild AD fared significantly better on tense/time reference than on grammatical aspect. The authors claimed that, at least in Greek, tense/time reference is less demanding than grammatical aspect in terms of processing resources, and they suggested that this was attributable to the fact that grammatical aspect is subjective (e.g., Comrie, 1976; Smith, 1997). Speakers of Greek, for example, always *choose* how to view past or future events; they view them in a perfective or in an imperfective way, and these choices are reflected in the use of perfective or imperfective grammatical aspect, respectively (for more details and examples, see *Background Information on Subject–Verb Agreement, Time Reference/Tense, and Grammatical Aspect*). When it comes to time reference, however, speakers’ decisions on the time frame to which they will refer are based on more objective criteria, such as location of event time in relation to speaking time (Fyndanis et al., 2018b). As noted by Fyndanis et al. (2018b, p. 1182), despite the fact that “constrained tasks employed to investigate aspect usually include adverbials that call for specific aspectual values […], it might be the case that the subjective component of aspectual representations is always ‘active’, rendering the decoding and encoding of aspect more costly than the decoding and encoding of tense […] Note that in sentence completion tasks the participant has to both decode the relevant features during the listening/comprehension part and encode them during the production part.”

Thus, it may be that the factor emphasized by IFIH (e.g., Fyndanis et al., 2012, 2018b), namely, ±involvement of integration processes, is relevant but interacts with other factors (e.g., ±subjectivity and ±involvement of inflectional alternations) in determining how taxing a given morphosyntactic category is. This interaction gives rise to a hierarchy of morphosyntactic categories reflecting the “amount” of demands they pose on the speaker’s processing system. The production pattern “grammatical aspect < time reference/tense < subject–verb agreement” (with the symbol < meaning “more prone to impairment”) reported for Greek by Fyndanis et al. (2012, 2013, 2018b) presumably reflects the hierarchy of these three morphosyntactic categories as per the processing load they are associated with. Given this, it is safe to assume that, for time reference to elicit worse performance than subject–verb agreement, the demands that the production of time reference pose on the processing system should exceed speakers’ available processing resources (for similar ideas, see Just and Carpenter, 1992). This assumption is relevant to the question why our MS participants were not impaired in tense/time reference. As mentioned above, Fyndanis et al’s. (2012, 2013, 2018b) aphasic and mild AD participants’ production performance on tense/time reference was better than that on grammatical aspect but worse than their production performance on subject–verb agreement. A possibility that cannot be ruled out is that individuals with agrammatic aphasia or mild AD have greater processing limitations than individuals with MS. Assuming that performance on the production of grammatical aspect is a sensitive index of the processing system’s efficiency, it should be noted that the group of eight Greek-speaking agrammatic participants reported by Fyndanis et al. (2018b) performed 40% correct on the production of grammatical aspect; the two Greek-speaking agrammatic participants reported in Fyndanis et al. (2012) performed 18 and 37% correct on the production of grammatical aspect; and the group of nine Greek-speaking participants with mild AD reported by Fyndanis et al. (2013) performed 37% correct on the production of grammatical aspect. While the aphasic and AD individuals reported in the studies above were severely impaired in the production of aspect, the individuals with MS who participated in the current study presented a mild-to-moderate impairment in the production of grammatical aspect, as the RRMS and SPMS groups fared 81 and 68% correctly, respectively. The above differences between Greek-speaking aphasic, AD, and MS groups in the level of performance on the production of grammatical aspect do not reflect task effects, as here we used the same sentence completion task as Fyndanis et al. (2018b), which was also similar to the completion tasks used in earlier studies on Greek agrammatic aphasia and AD by Fyndanis et al. (2012, 2013). One could argue, therefore, that the processing system of the MS individuals who participated in the present study was still efficient enough to handle time reference/tense.

Note that not only Fyndanis et al. (2012, 2018b) but also Varlokosta et al. (2006) and Nanousi et al. (2006) found grammatical aspect to be more impaired than subject–verb agreement in Greek (agrammatic) aphasia (but see Protopapas et al., 2016). Therefore, similar patterns emerge across different pathologies (aphasia, AD, and MD) and healthy speakers, with the most robust finding being that, at least in Greek, grammatical aspect is harder to produce than subject–verb agreement. On the assumption that, in both neurologically healthy and brain-damaged speakers, accuracy performance on the production of demanding verb-related morphosyntactic categories such as grammatical aspect largely depends on the available processing resources, which are presumably reflected in verbal WM capacity and/or processing speed, and given that there are individual differences in verbal WM capacity and processing speed in both healthy speakers and neurological populations, it is not surprising that the pattern “grammatical aspect < subject–verb agreement” consistently emerges in different populations.

That the same pattern of morphosyntactic impairment emerges in different pathologies characterized by lesions or atrophy in different brain regions is not surprising either, as WM (which is closely related to the cognitive construct of processing resources) is subserved by a broadly distributed neuronal network, involving both anterior and posterior portions of the brain, including Broca’s area (which is usually affected in agrammatic aphasia), the basal ganglia (often affected in MS; Chiang et al., 2019), and the medial temporal lobe (which is where the atrophy starts in AD) (e.g., Gabeza et al., 2002; McNab and Klingberg, 2008).

To summarize, based on the findings of studies on the production of subject–verb agreement, time reference/tense, and grammatical aspect in Greek-speaking healthy aging (Fyndanis et al., 2018b; Masoura et al., 2018), aphasia (Nanousi et al., 2006; Varlokosta et al., 2006; Fyndanis et al., 2012, 2018b), mild AD (Fyndanis et al., 2013), and MS (present study), we argue that, since subject–verb agreement, time reference, and grammatical aspect have intrinsically different properties (e.g., ±presence of interpretable feature, ±involvement of integration processes, and ±subjectivity), they pose differential demands on the speaker’s processing system. Thus, the most demanding category (i.e., grammatical aspect) taxes the speaker’s processing system, and speakers with a less efficient processing system perform worse on the production of this category than on the production of less demanding categories (e.g., subject–verb agreement). Therefore, this pattern of performance stems from the combination of the differential demands that subject–verb agreement, time reference, and grammatical aspect pose on the speaker’s processing system and the individual differences in processing efficiency observed in both pathological and healthy speakers. Worse accuracy performance on aspect than on agreement at the group level should be driven by participants with a less efficient processing system. Participants with a very efficient processing system would be expected to have ceiling accuracy performance on both agreement and aspect. Even in such participants, however, the differential demands that these two morphosyntactic categories make to the processing system should be reflected in dependent variables more sensitive than accuracy, such as reaction times. Hence, we argue that the emerging patterns of performance on verb-related morphosyntactic production depend on three factors that act in synergy: (1) intrinsically different properties (and differential demands) of morphosyntactic categories under investigation; (2) presence of sufficiently large variability in processing efficiency across participants; and (3) dependent variable being used (e.g., accuracy or reaction times). Importantly, (degree or level of) processing efficiency appears to play an important role in the production of verb inflection not only because it may shape the pattern of morphosyntactic performance but also because quantitative differences between brain-damaged and healthy control groups in morphosyntactic production might partly stem from between-group differences in processing efficiency (Fyndanis et al., 2018b). Processing efficiency relevant to verb-related morphosyntactic production may be reflected in verbal WM capacity (op. cit.) and/or processing speed. Nevertheless, we have to acknowledge that, although verbal WM is the cognitive system that has attracted the attention of most scholars who investigate morphosyntactic impairments, there is still uncertainty on the nature of processing efficiency that is relevant to verb-related morphosyntactic production, and on the exact cognitive mechanism that is critically involved in the production of demanding morphosyntactic categories such as grammatical aspect. Is it only verbal WM that matters, or other cognitive capacities such as processing speed, inhibition, and updating also play a role?

### Alternative Accounts

Faroqi-Shah and Thompson (2007) suggested that tense and (grammatical) aspect might be affected in agrammatic aphasia because of an impairment in the cognitive process of *encoding* tense-related or aspect-related abstract, prephonological features (e.g., +PAST and −PERFECTIVE) and/or in *retrieving* the corresponding verb form or verb inflection. This is a plausible explanation for the pattern of performance exhibited by our participants with MS. The sentence completion task we used, however, tapped into aspect-related encoding and retrieval processes to a similar extent. This is so because, to perform this task, participants had to both *encode* an abstract, prephonological feature (+PERFECTIVE or −PERFECTIVE) other than that encoded in the verb form of the SS, *and* to *retrieve* a corresponding verb form/inflection. Hence, our design does not allow us to detect the exact nature of the deficit in grammatical aspect that emerged in Greek MS. (For suggestions on how to tease apart encoding from retrieval processes, see Fyndanis et al., 2018a).

Another plausible explanation could be inspired by the tense underspecification hypothesis (TUH) (Wenzlaff and Clahsen, 2004, 2005), proposed to account for selective patterns of morphosyntactic impairment in Broca’s/agrammatic aphasia. The TUH posits that, while the agreement and grammatical mood features are well preserved in the grammars of persons with Broca’s/agrammatic aphasia, the tense feature is underspecified (i.e., it has no value). In the same vein, one could assume that, while the agreement and tense features are intact in the grammars of Greek-speaking individuals with MS, the aspect feature is intermittently underspecified, resulting in sporadic “random retrieval” of verb forms/inflections in the sense that, since no abstract aspectual value has been specified, no such value could guide the retrieval of a particular verb form/inflection. Again, one could not rule out this possibility. However, the present design does not allow us to pinpoint the source of our MS participants’ difficulty producing grammatical aspect.

The observed pattern of morphosyntactic production cannot be accounted for by accounts employing the minimalist construct of *Merge* (e.g., Chomsky, 1995, 2000, 2001) or the generative construct of *syntactic hierarchy/tree*. For example, Hagiwara (1995) argued that the higher a node/category in the syntactic hierarchy, the costlier it is computationally, because the operation *Merge* has to be implemented more times compared with categories that are located lower in the syntactic hierarchy. Nevertheless, grammatical aspect is low in the syntactic hierarchy of Greek (just above the verb phrase; e.g., Alexiadou and Anagnostopoulou, 1998; Philippaki-Warburton, 1998). Therefore, it is not computationally costly to project the aspect node in Greek.

The tree pruning hypothesis (TPH) (Friedmann and Grodzinsky, 1997), proposed to capture data from aphasia, could not be extended to account for our results either. The TPH states that, in agrammatic aphasia, the syntactic tree (hierarchy) is usually pruned at a relatively high position, usually at the tense node, with all the nodes above the pruning site being inaccessible and all the nodes below being well-preserved. Our participants with MS performed well on tense/time reference. Even if we assume that their syntactic tree was pruned at the aspect node (that is, at a low position in the syntactic hierarchy of Greek), the fact that our MS participants performed very well on morphosyntactic features hosted by nodes situated above aspect (such as agreement and tense features) is not consistent with the predictions of the TPH.

### Limitations of the Current Study

We acknowledge two main limitations of this study. The first one relates to the fact that the participants with MS and the healthy controls have not completed a thorough cognitive assessment. In fact, only the MS participants completed a brief cognitive assessment (BICAMS; Langdon et al., 2012; Polychroniadou et al., 2016). This limits the possibility to draw solid conclusions about the role of cognition in morphosyntactic difficulties. The second limitation relates to the relatively small samples of participants with RRMS and SPMS. Small samples of participants make it harder to detect between-group and/or within-group significant differences. For instance, the lack of significant difference between the two MS groups in morphosyntactic performance does not provide evidence for the absence of a difference (which can still be present and not detected), nor does the lack of significant difference between time reference/tense and subject–verb agreement (in either of the two MS groups) constitute evidence for the absence of a difference between these two morphosyntactic categories. If these two MS groups had been larger, a significant between-group difference in morphosyntactic performance and/or a significant difference between time reference/tense and subject–verb agreement might have been found.

### Future Directions

In future research, we plan to address which cognitive systems are critically involved in verb-related morphosyntactic production (with a special focus on the production of grammatical aspect) and what is the exact role of each cognitive capacity in the production of verb inflection. To this end, we intend to measure a number of cognitive abilities of individuals with MS or other neurological conditions, such as verbal WM, visuospatial WM, verbal STM, visuospatial STM, processing speed, and components of executive functioning such as inhibition and updating. These cognitive capacities are related to each other, but it is not clear if all of them play a role in verb-related morphosyntactic production. Such a broad cognitive testing will enable us to shed more light on the relationship between cognitive capacities and verb-related morphosyntactic production, and on the underlying causes of impaired morphosyntactic production in MS and other neurological conditions.

Future research should also investigate whether grammatical aspect, the most demanding and vulnerable verb-related morphosyntactic category, could serve as a linguistic clinical marker that could distinguish between different clinical phenotypes of MS. In the present study, the SPMS group performed worse (albeit not significantly so) than the RRMS group on grammatical aspect. If increasing the sample size of RRMS and SPMS participants resulted in significant between-MS subgroup differences in grammatical aspect, this would be evidence that this morphosyntactic category could serve as a clinical marker distinguishing between these two phenotypes of MS.

Lastly, we plan to investigate the neural substrate of impaired verb-related morphosyntactic production in MS by obtaining neuroimaging data and exploring the associations between affected brain areas/neuronal networks and performance on tasks tapping into verb-related morphosyntactic production. If the neuroimaging data show atrophy in the basal ganglia of our MS participants (which would be consistent with Chiang et al’s., 2019, findings), such a study will enable us to test Ullman’s (2001, 2004) claim that rule-based morphological processes such as affixation involved in the production of regular past-tensed verbs (e.g., walk–walk*ed*) is supported by a “procedural” system (procedural memory), which is localized in the basal ganglia, regions of the frontal and parietal lobes, and the dentate nucleus of the cerebellum.

## Conclusion

The main finding of this study is that verb-related morphosyntactic production can be impaired in MS, and this impairment can be observed in both RRMS and SPMS. In the context of Greek MS, grammatical aspect was found to be the most severely affected morphosyntactic category. Since worse performance on grammatical aspect than on subject–verb agreement has consistently been found not only in different types of pathological language such as that in MS (present study), mild AD (e.g., Fyndanis et al., 2013), and stroke-induced aphasia (e.g., Nanousi et al., 2006; Varlokosta et al., 2006; Fyndanis et al., 2012, 2018b) but also in neurologically healthy speakers (e.g., Fyndanis et al., 2018b), it seems that pathology exacerbates patterns found in healthy speakers, which is consistent with Dick et al. (2001) and Miyake et al. (1994). Furthermore, we argue that, because of their intrinsically different linguistic properties, grammatical aspect, time reference, and subject–verb agreement pose differential demands on the speaker’s processing system, and whenever the demands posed by the most taxing category (i.e., aspect) exceed the capacity of the processing system, the speaker will likely produce a verb form that does not encode the target feature (i.e., *perfective* or *imperfective* aspect). Finally, given the production of grammatical aspect falls into the broader domain of “expressive language,” and since the majority of individuals with MS have self-reported a language impairment affecting expressive language (among other linguistic domains), and also experienced a reduced quality of life compared to MS individuals without language impairment (El-Wahsh et al., 2020), our MS participants’ impairment in the production of grammatical aspect may be clinically relevant.

## Data Availability Statement

The datasets generated for this study are available on request to the corresponding author.

## Ethics Statement

The study was reviewed and approved by the Ethics Committee of the University Hospital of Ioannina, Greece. The participants provided their written informed consent to take part in this study.

## Author Contributions

VF, LM, GN, and ED contributed to the conception and design of the study. LM, GN, ED, MM, MP, and AN contributed to participant recruitment and/or data collection and transcription. SM and VF analyzed the data. VF, SM, LM, and GN wrote the first draft of the manuscript. All authors contributed to manuscript revision, and approved the submitted version.

## Conflict of Interest

The authors declare that the research was conducted in the absence of any commercial or financial relationships that could be construed as a potential conflict of interest.

## Footnotes

1 This section was based on Fyndanis (2019) and work cited therein.

3 The production of verb inflection has been found to be impaired not only in AD but also in primary progressive aphasia; for a systematic review covering both AD and primary progressive aphasia, see Auclair-Ouellet (2015).

4 Similar results have been reported by Masoura et al. (2018), who investigated the role of WM, short-term memory (STM), and inhibition in verb-related morphosyntactic production in healthy middle-aged and older Greek-speaking participants.

## Abbreviations

AD: Alzheimer’s disease
BICAMS: Brief International Cognitive Assessment for MS
BMVT-R: Brief Visuospatial Memory Test-Revised
EDSS: Expanded Disability Status Scale
GVLT: Greek Verbal Learning Test
IFIH: Interpretable Features Impairment Hypothesis
MMSE: Mini Mental State Examination
MS: multiple sclerosis
PWA: persons with aphasia
RRMS: relapsing-remitting multiple sclerosis
SDMT: Symbol Digit Modalities Test
SPMS: secondary progressive multiple sclerosis
SS: source sentence
STM: short-term memory
TPH: tree pruning hypothesis
TS: target sentence
TUH: tense underspecification hypothesis
WM: working memory.
