# Supplementary material for: Impaired Verb-Related Morphosyntactic Production in Multiple Sclerosis: Evidence From Greek
Source: Front Psychol. 2020 Aug 27;11:2051. doi: 10.3389/fpsyg.2020.02051 (PMC7481395; doi:10.3389/fpsyg.2020.02051)
Supplement: Supplementary file 1 [file Data_Sheet_1.docx]

**Impaired verb-related morphosyntactic production in multiple sclerosis: Evidence from Greek**

**Supplementary Material S1**

Descriptions of SDMT, BMVT-R, and GVLT

The SDMT consists of a key that includes nine pairs of symbols and numbers (1-9), and of 8 different sequences of symbols, with each sequence consisting of 15 symbols. Only the key’s symbols are included in these sequences. Participants are instructed to write down below each symbol the number that it is associated with (substitution) as fast as possible. The first sequence of symbols was used in the practice session. Total score is derived from the number of correct answers/substitutions within 90 seconds. Maximum correct score is 110.

In the BVMT-R, the participant is presented with a page that includes six line drawings for 10 seconds, and asked to memorize this page. Subsequently, they are given a blank page and a pencil or pen, and are instructed to draw the line drawings they memorized previously as accurately as possible and in the same location as they appeared on the memorized page. Depending on accuracy and location, each line drawing is scored with zero, one or two points. The procedure is repeated three times. Total score is the sum of the individual scores allocated for the line drawings in the three trials (Polychroniadou et al., 2016).

The GVLT provides three alternate forms with satisfactory psychometric properties. In the present study we used Form A (GVLT A). GVLT consists of a list of 16 words, which the participant must learn in five consecutive but independent learning trials. Total score is derived from the total number of words recalled in all five trials (Vlahou et al., 2013; Polychroniadou et al., 2016, p. 69). GVLT provides the same number of words and trials as used in the California Verbal Learning Test – II, which is used in the original BICAMS (Langton et al., 2012). Words used in the GVLT have been culturally adapted for the Greek population.

**Supplementary Material – Table S1**. Generalized linear mixed-effects model on accuracy fitted to the unified MS dataset. The model included the additive effect of Morphosyntactic Condition and List, the interaction between the two, Subjects and Items as random effects, and Morphosyntactic Condition as by-Subject random slope. The symbol * indicates significant effects.

| Term | Estimate | Std. Error | z value | Pr(>\|z\|) |
| --- | --- | --- | --- | --- |
| Intercept (List=List 1; Morphosyntactic Cond.=Agreement) | 5.180 | 1.144 | 4.527 | <.001* |
| Morphosyntactic Cond.=Aspect | -3.090 | 1.051 | -2.939 | .003* |
| Morphosyntactic Cond.=Time Ref. | -1.598 | 1.511 | -1.058 | .290 |
| List=List 2 | 0.005 | 0.703 | 0.008 | .994 |
| Morphosyntactic Cond.=Aspect :  List=List 2 | -0.171 | 0.638 | -0.268 | .789 |
| Morphosyntactic Cond.=Time Ref. : List=List 2 | 1.580 | 0.994 | 1.589 | .112 |
| Intercept (List=List 1; Morphosyntactic Cond.=Aspect) | 2.091 | 0.939 | 2.226 | .026* |
| Morphosyntactic Cond.= Agreement | 3.090 | 1.053 | 2.935 | .003* |
| Morphosyntactic Cond.=Time Ref. | 1.492 | 1.504 | 0.992 | .321 |
| List=List 2 | -0.166 | 0.595 | -0.278 | .781 |
| Morphosyntactic Cond.=Agreement : List=List 2 | 0.171 | 0.639 | 0.267 | .789 |
| Morphosyntactic Cond.=Time Ref. : List=List 2 | 1.750 | 1.030 | 1.699 | .089 |
